# Control of stripe rust of wheat using indigenous endophytic bacteria at seedling and adult plant stage

**DOI:** 10.1038/s41598-021-93939-6

**Published:** 2021-07-14

**Authors:** Tehmina Kiani, Farrakh Mehboob, Muhammad Zeeshan Hyder, Zainy Zainy, Liangsheng Xu, Lili Huang, Sumaira Farrakh

**Affiliations:** 1 Department of Biosciences, COMSATS University Islamabad, Islamabad, Pakistan; 2 National Agriculture Research Center, Islamabad, Pakistan; 3 College of Plant Protection, Northwest A&F University, Yangling, China

**Keywords:** Microbiology, Plant sciences

## Abstract

Stripe rust (caused *by Puccinia striiformis tritici*) is one of the most devastating diseases of wheat. The most effective ways to control stripe rust are the use of resistant cultivars and the timely use of an appropriate dose of fungicide. However, the changing nature of rust pathogen outwits the use of resistant cultivars, and the use of a fungicide is associated with environmental problems. To control the disease without sacrificing the environment, we screened 16 endophytic bacteria, which were isolated from stripe rust-resistant wheat cultivars in our previous study, for their biocontrol potential. A total of 5 bacterial strains *Serratia marcescens* 3A, *Bacillus megaterium* 6A, *Paneibacillus xylanexedens* 7A, *Bacillus subtilis* 11A, and *Staphyloccus agentis* 15A showed significant inhibition of *Puccinia striiformis* f. sp. *tritici* (Pst) urediniospores germination. Two formulations i.e., fermented liquid with bacterial cell (FLBC) and fermented liquid without bacterial cells (FL) of each bacterial strain, were evaluated against the urediniospores germination. Formulations of five selected endophytic bacteria strains significantly inhibited the uredinioospores germination in the lab experiments. It was further confirmed on seedlings of Pakistani susceptible wheat cultivar Inqilab-91 in the greenhouse, as well as in semi-field conditions. FLBC and FL formulations applied 24 h before *Pst* inoculation (hbi) displayed a protective mode. The efficacy of FLBC was between 34.45 and 87.77%, while the efficacy of FL was between 39.27 and 85.16% when applied 24 hbi. The inoculated wheat cultivar Inqilab-91 was also tested under semi-field conditions during the 2017–2018 cropping season at the adult plant stage. The strains *Bacillus megaterium* 6A and *Paneibacillus xylanexedens* 7A alone significantly reduced the disease severity of stripe rust with the efficacy of 65.16% and 61.11% for the FLBC in protective effect, while 46.07% and 44.47% in curative effect, respectively. Inoculated seedlings of Inqilab-91 showed higher activities of antioxidant enzymes, superoxide dismutase (SOD), peroxidase (POD), polyphenol oxidase (PPO), and phenylalanine ammonia-lyase (PAL). The treated seedlings also showed higher expressions of pathogenesis-related (PR) protein genes, antifungal protein (PR-1), β-1,3-endoglucanases (PR-2), endochitinases (PR-4), peroxidase (PR-9), and ribonuclease-like proteins (PR-10). These results indicated that endophytic bacteria have the biocontrol potential, which can be used to manage stripe rust disease. High production antioxidant enzymes, as well as high expression of PR protein genes, might be crucial in triggering the host defense mechanism against *Pst*.

## Introduction

The increasing world population, urbanization, rising incomes, and change in eating preferences derive a rapid increase in global wheat consumption. By 2050, a 60% higher demand for wheat than today is forecasted. This vast challenge may be accomplished by utilizing more land, water, and labor, effectively using fertilizers, and controlling pathogens, which result in detrimental yield and quality losses worldwide^1,2^. Among pathogens, rust diseases are one of the main constraints in wheat production.

In Pakistan, it is the single most important food crop, contributing 72% of Pakistan's daily caloric intake with per capita consumption of around 124 kg per year, one of the highest in the world. It is cultivated by 80% of farmers on approximately 9 million hectares of land (40% of the total cultivated land). A total of 5.8 m ha (70%) of the total area under wheat cultivation is prone to stripe rust^3^. So far in Pakistan, four major stripe rust epidemics have been observed, with intensity exceeding 20% during the years 1973 (35%), 1978 (55%), 1995 (37.5%), and 2005 (20%). Stripe rust intensity has never fallen below 8% in Pakistani history since the 1950s^4^. During the year 2019, it was reported that the intensity of stripe rust in Pakistan exceeded more than 30%, causing, severe damage to wheat production. Currently, it prevails across all wheat-growing areas from the north to the south, which are areas of low and high temperatures, respectively, in Pakistan^5^.

Several measures can be taken to overcome stripe rust. It can be controlled by planting on time, using a mixture of various cultivars, appropriate fertilization and irrigation, timely use of fungicides, accurate formulations, and use of resistant cultivars^6^. Some measures are difficult to execute due to their laborious and time-consuming restraints, some of which are impracticable for several wheat-producing areas, and the use of a fungicide is not environmentally friendly. Since rust is equipped with means of rapid recombination, it can become resistant against the fungicide. In Pakistan, use of resistant cultivars is an affordable control measure. The main drawback with this measure is that resistance of cultivars to stripe rust could be easily overcome by new virulent *Pst* races^6^. Pathologists are now focusing on the use of biocontrol agents to combat rust. A few cases of successful biocontrol of stripe rust or suppression of stripe rust urediniospores have been reported by using plant extracts or endophytic organisms.

Endophytes are microorganisms that can be easily isolated from the inner parts of plants or surface disinfected tissues. Moreover, their ecological niche, analogous to the phytopathogens, favor them as the most suitable candidate for biocontrol agents. Microbes have been used for the control of rust diseases. Isolates of *Bacillus subtilis*, *B. cereus* (subsp. mycoides), *B. thuringiensis*, and Erwinia ananas pv. *uredovora* were found effective in controlling rust on beans^7^. *Pantoea agglomerans* B1, and *Stenotrophomonas maltophilia* C3, were reported effective in reducing bean rust severity^8^, while *Bacillus sp. isolate* B157 was reported as a potential biocontrol agent for coffee rust^9^. Although there is a long history of controlling rust pathogens using endophytic bacteria, biological control of *Punnicia striiformis tritici* was reported for the first time by^10^. In their study, endophytic *Bacillus subtilis* strain E1R-j, which was isolated from wheat roots, reduced the disease severity of wheat stripe rust in both greenhouse and field experiments. In another study, the JD204 strain of *Pseudomonas putida,* which was isolated from the roots of winter wheat plants, was reported to induce resistance in cultivar by the over-expression of genes encoding the resistance-related enzymes^11^. *Bacillus subtilis* strain QST 713 suspension concentrate (SerenadeASO) was investigated for its potential of stripe rust control in winter wheat field trials. SerenadeASO reduced the severity of yellow rust significantly at the early growth stage under moderate disease pressure. Under high disease pressure reductions were more variable^12^. Wang^13^ obtained an endophytic *Pseudomonas aurantiaca* strain and found it effective in suppressing wheat leaf rust. Flaishman et al.^14^ reported recombinant *P. putida* strains capable of suppressing wheat leaf rust. Recently, combinations of *Trichoderma* spp. and arbuscular mycorrhizal (AM) fungi were also reported to lower disease incidence of wheat stem rust^15^.

In the current study, sixteen indigenous endophytic bacteria, previously isolated from stripe rust-resistant wheat cultivars^16^, were explored for their possible potential as a biocontrol agent against stripe rust.

## Results

### In vitro inhibition efficiency of bacterial strains against *Pst*

Sixteen bacterial strains isolated from the resistant wheat cultivars were tested in-vitro for antagonistic effects in 0.5% water agar against *Pst* CYR-31. Out of 16, only 14 showed inhibition of spore germination. Among these, nine strains showed the inhibition efficacy greater than 20% but less than 50% in both types of bacterial formulations. However, the five strains (*Serratia marcescens 3A, Bacillus megaterium 6A, Paneibacillus xylanexedens 7A, Bacillus subtilis11A,* and *Staphylococcus agentis 15A*) showed a higher inhibition rate of > 50% in either FL or FLBC formulations.

**Figure 1:**
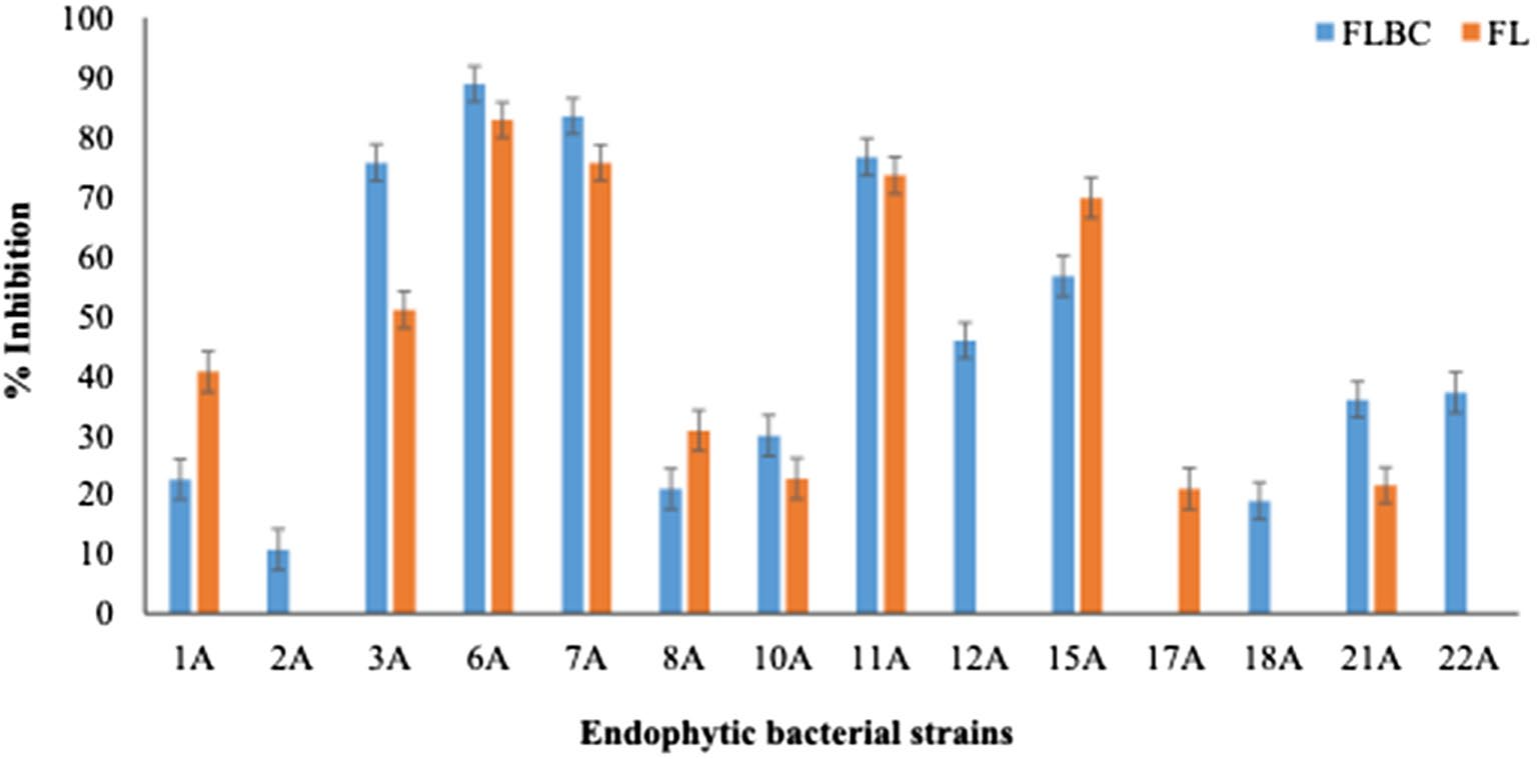
Inhibition efficiency of bacterial endophytes isolated from the resistant wheat cultivars against *Pst. Serratia marcescens (3A), Bacillus megaterium (6A), Paenibacillus xylenexedens (7A), Bacillus subtilus (11A), Staphylococcus agentis (15A)*.

**Figure 2:**
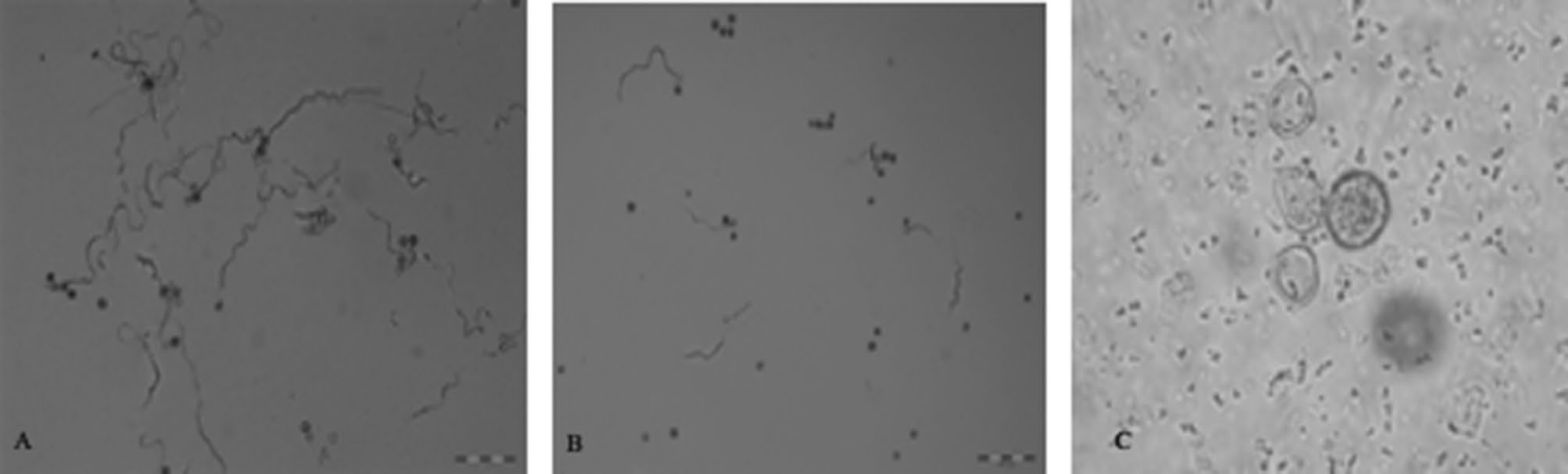
Germination of Urediniospores: **A**) Control, **B**) FL of *Paenibacillus xylanexedens* 7A, **C**) FLBC of *Paenibacillus xylanexedens* 7A. Gennioation of Urediospores: (**A**) Coolrol, (**B**) FL of *Paneibacillus xylanexedens* 7A, (**C**) FLBC of *Paneibacillus xylanexedens 7A*.

The FLBC of *Serratia marcescens* 3A, *Bacillus megaterium* 6A, *Paneibacillus xylanexedens* 7A, and *Bacillus subtilis* 11A showed higher inhibition of the urediniospore germination than FL. However, only FL of *Staphyloccus agentis 15A* showed higher inhibition of urediniospores than its FLBC (Figs. 1 and 2).

## Greenhouse experiment

### Antagonist effects of FLBC and FL formulations on Inqilab-91 challenged with CYR-31 at seedling stage

The bacterial formulation FLBC sprayed at 24 h before (protective effect) and 24 h after (curative effect) *Pst* inoculation, significantly reduced the disease severity in Inqilab-91. When the seedlings were treated 24 hbi with FLBC formulation, *P. xylanexedens 7A* showed a control efficacy of 87.77% and *B. megaterium 6A* showed a controlled efficacy of 86.94%. The bacterial treatments of *B. subtilus 11A showed* 73.19%. However, *S. marcescens 3A* and *S. agentis 15A* exhibited control efficacy of only 52.04 and 45.45% at 24 hbi of *Pst.*

In curative effect, FLBC of *P. xylanexedens 7A* showed 84.47% and *B. megaterium 6A* showed 82.07%, significantly higher control efficacy when sprayed to seedlings 24 hai.

**Figure 3:**
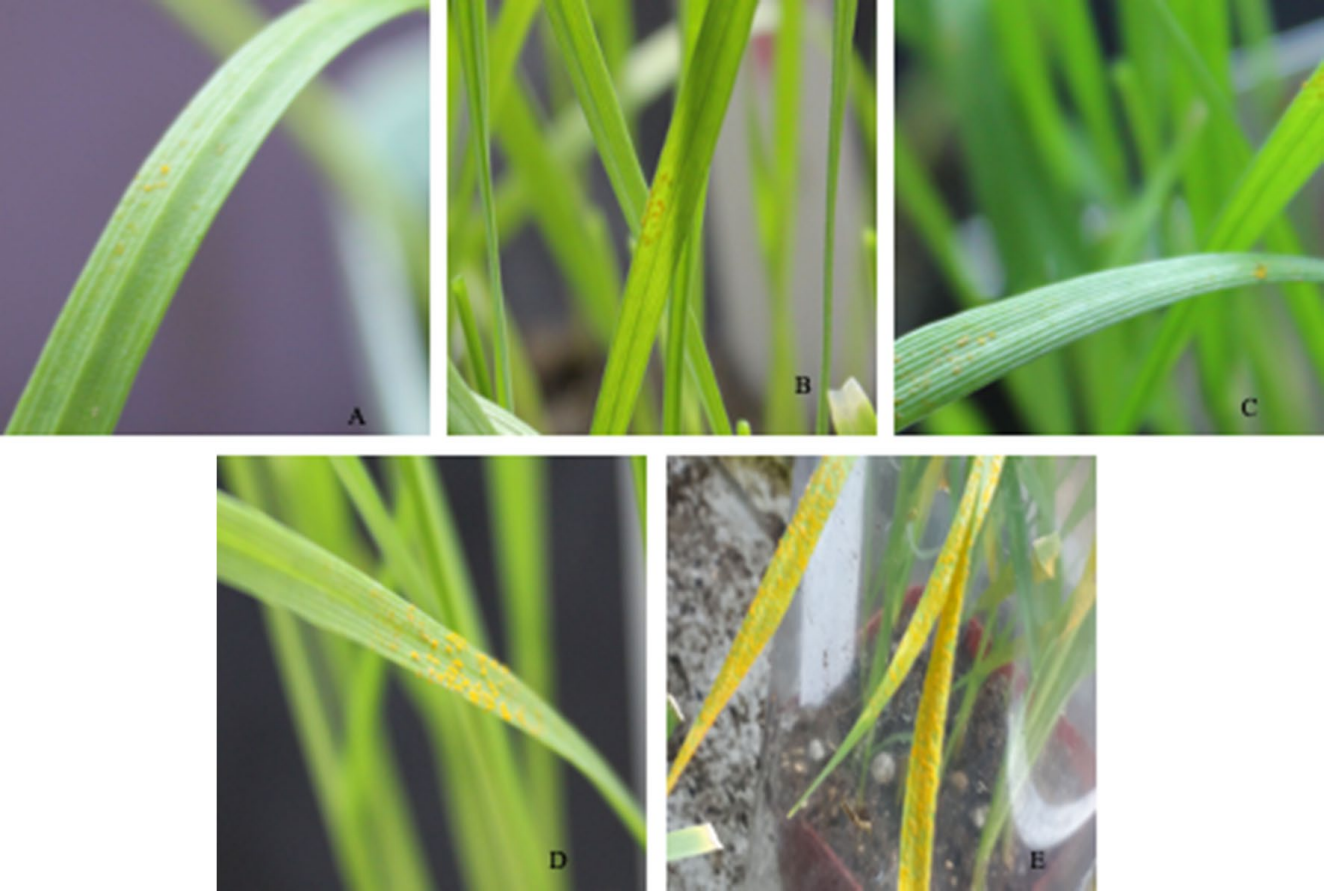
Sporulation of susceptible wheat cultivar Inqilab-91 seedling leaves 14 days after co-inoculation of *Puccinia striiformis f. sp. tritici (Pst)* and bacterial treatments. (**A**) Leaf treated with inoculation *Paneibacillus xylanexedens 7A*, in protective treatement, (**B**) in curative treatment. (**C**) Leaves treated *Bacillus megaterium 6A* in protective treatment, (**D**) in curative treatment, (**E**) leaves treated with LB only.

When the wheat seedlings were inoculated with FL formulation 24 hbi (protective effect), maximum control efficacy of 85.35% was observed for *B. megaterium 6A* followed by *B. subtilus 11A *(82.16%)* and P. xylanexedens 7A* (75.28%). Similarly, in curative effect, maximum control efficacies of 77.65% and 65.65% were observed with the FL of *B. megaterium 6A* and *B. subtilus* 11A when sprayed to seedlings 24 hai (Table 1 and Fig. 3).

**Table 1:** Control efficacy (%) of selected bacterial strains in Inqilab-91, using fermented liquid with bacterial cell suspension (FLBC) and fermented liquid without bacterial cell suspension (FL) for Protective and Curative effects at seedling stage.

Endophytic bacteria	Protective effect (24 hbi)					Curative effect (24 hai)				
	Control	FLBC		FL		Control	FLBC		FL	
	Severity	Severity	Control efficacy	Severity	Control efficacy	Severity	Severity	Control efficacy	Severity	Control efficacy
*Paenibacillus xylanexedens 7A*	75.21 ± 0.89	11.47 ± 1.7Aa	87.77	23.17 ± 2.8Ba	75.28	75.01 ± 1.89	14.56 ± 2.0Aa	84.47	35.67 ± 2.0Aa	48.03
*Serratia marcescens 3A*		51.15 ± 1.2Ab	45.45	56.93 ± 2.0Bbe	39.27		54.23 ± 2.0Ab	42.17	69.43 ± 5.6Bbd	29.92
*Bacillus subtilus 11A*		25.13 ± 1.8Ac	73.19	16.76 ± 1.9Ba	82.16		31.99 ± 1.5Ac	65.89	41.72 ± 7.5Aac	65.65
*Bacillus megaterium 6A*		12.23 ± 1.7Aa	86.94	13.72c ± 1.7Ac	85.35		16.81 ± 1.9Aa	82.07	38.69 ± 6.0Ba	77.65
*Staphylococcus agentis 15A*		44.96 ± 3.0Ad	52.04	53.46 ± 2.0Bde	42.97		59.87 ± 3.0Ad	36.15	65.28 ± 5.2Bcd	35.28

Data presented here are mean ± standard deviation.

Means were compared with Tukey HSD (p ≤ 0.01, 0.05).

Treatments were conducted by spraying different liquids with or without bacterial cells. *Hbi* hours before inoculation, *hai* hours after inoculation.

Stripe rust severity was recorded 14 days after *Pst* inoculation.

The data are the means of ten plants per treatment with three replications. Low case letters showed comparisons among the 5 bacterial treatments (in column) and capital letters showed comparisons among bacterial formulations FLBC and FL (in rows).

### Microscopic examination of rust and endophytic bacterial interaction

**Figure 4:**
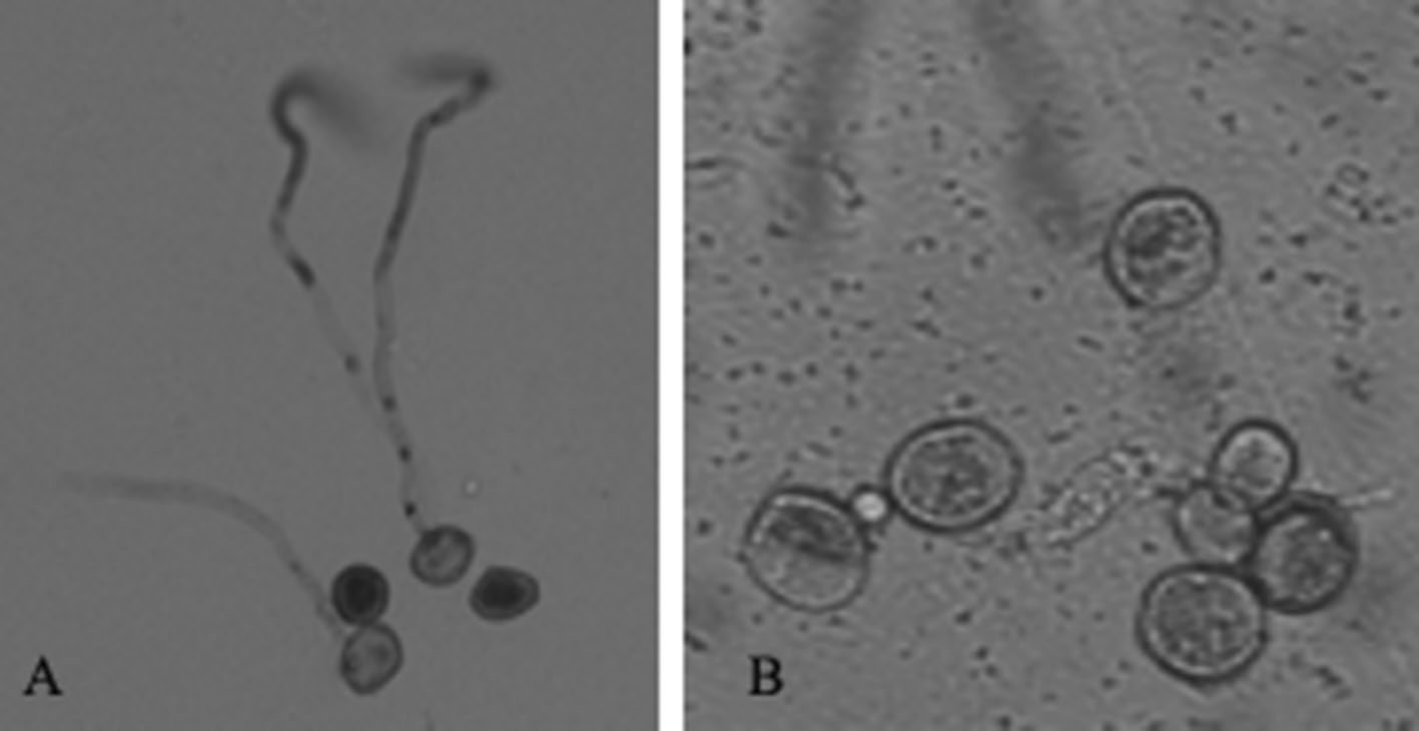
Optical microscopic study of effect of biocontrol bacteria and the *Pst *on change in morphology. (**A**) indicate ruptured or re1eased protoplasm (FL) on the agar plates. (**B**) Show urediospores remained non­germinated by swelling (FLBC).

Microscopic observations using an optical microscope showed that germination of urediniospores was significantly affected by FLBC and FL formulations (Fig. 4).

### Scanning electron microscope (SEM) observation

**Figure 5:**
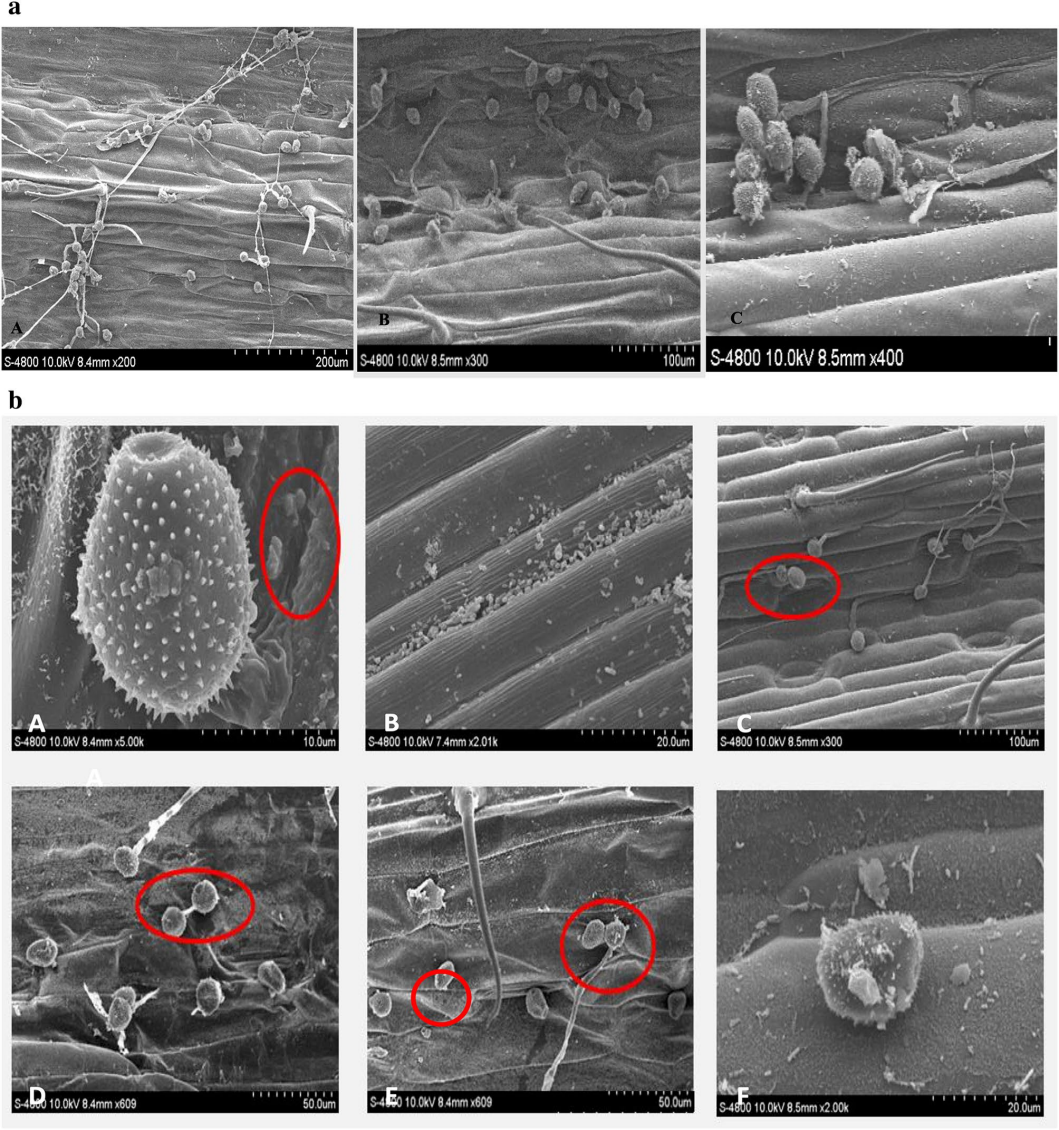
(**a**) Scanning electron microscopic observations of the effects of *Pst *and selected biological control agents (BCAs). A: Germination of *Pst *conttrol (without bacterial inculation). B: Effect of FLBC on the growth of *Pst* of 24 h after bacterial treatments. C: Effect of FLBC on the growth of *Pst *(24 hbi) (non-germinated spores). (**b**) Morphological changes observed during the scanning electron microscopy showing the effect of *Pst on *BCA. A: Endophytic bacteria inhibiting the germination of urediospore; B: abundance of endophytic bacteria; C: Swelling of non-germinated urediospore; D: dimer formation; E, F: change in morphology of non­germinated urediospore from round to oval.

Normal germination of urediniospores was observed by SEM analysis on control leaves treated with LB media and water. However, several morphological changes were observed in FL and FLBC treated urediniospores. The treated urediniospores either remained non-germinated, ruptured, shrieked, or malformed (Fig. 5a,b).

### Molecular confirmation of inoculated endophytic bacteria in leaves

The presence of endophytic bacteria in the inoculated leaves was further confirmed through 16s rRNA gene amplification using universal primers and RFLP analysis using the same enzymes as used in the study^16^.

### Antagonistic effects of bacterial strains on stripe rust at adult plant stage in pots under semi- field conditions

Both formulations FLBC and FL showed significantly high control efficacy under semi-field conditions during the 2017–2018 growing season (Table 2). However, in pot experiments, the overall efficacies of these bacterial strains were lower than in the greenhouse.

**Table 2:** Control efficacy of FLBC and FL treatments of selected bacterial strains on *Pst *germination under semi-field conditions. Bulk *Pst* inoculum inoculated on Inqilab-91 tested in semi-field conditions.

Endophytic bacteria	Protective effect (24 hbi)					Curative effect (24 hai)				
	Control	FLBC		FL		Control	FLBC		FL	
	Severity	Severity	Control efficacy	Severity	Control efficacy	Severity	Severity	Control efficacy	Severity	Control efficacy
*Paenibacillus xylanexedens 7A*	75.21 ± 0.89	36.45 ± 2.5Aa	61.11	35.67 ± 2.0Aa	61.95	75.01 ± 1.89	52.05 ± 2.5Aa	44.47	63.38 ± 3.0Ba	21.36
*Serratia marcescens 3A*		63.62 ± 7.9Abd	32.12	69.43 ± 5.6Bbd	25.93		59.12 ± 7.8Ab	15.51	73.36 ± 5.2Ba	5.92
*Bacillus subtilus 11A*		41.38 ± 5.3Aa	55.85	41.72 ± 7.5Aac	55.49		69.67 ± 5.3Ac	25.89	57.34 ± 7.5Bb	38.99
*Bacillus megaterium 6A*		32.65 ± 11.1Aa	65.16	38.69 ± 6.0Ba	58.68		50.56 ± 12.1Aa	46.07	46.04 ± 6.0Ac	50.99
*Staphylococcus agentis 15A*		62.45 ± 4.2Acd	22.71	65.28 ± 5.2Bcd	29.64		64.20 ± 4.2Ad	9.48	65.87 ± 5.2Aa	8.61

Data presented here are mean ± standard deviation.

Means were compared with Tukey HSD (p ≤ 0.01, 0.05).

The data are the means of 10 plants per treatment with three replications. Low case letters showed comparisons among the 5 bacterial treatments and capital letters showed comparisons among bacterial formulations (FLBC and FL).

### Biocontrol efficacy of FLBC in semi-field natural environment

The control efficacy of FLBC in 24 hbi was significantly higher than 24 hai. FLBC treatment of bacterial strains *B. megaterium 6A* showed maximum reduction in disease severity (65.16%) followed by *P. xylanexedens 7A* (61.11%) and *B. subtilus 11A* (55.85) by showing protective effect (24 hbi). In curative effect, significantly higher disease reduction was observed in *B. megaterium 6A* 46.07% and *P. xylanexedens 7A* 44.47%, followed by *B. subtilus 11A* 25.89% (Table 2).

### Biocontrol efficacy of FL in semi-field natural environment

Bacterial strains of *P. xylanexedens 7A* (61.95%), *B. megaterium 6A *(58.6%), and *B. subtilus 11A* (55.4%) showed maximum reduction in disease severity when FL formulation was sprayed 24 hai.

In the case of the curative effect of the FL formulation, the maximum disease reduction was observed when the FL formulation of *B. megaterium 6A* (50.99%), *B. subtilus 11A* (38.99%), and *P. xylanexedens 7A* (21.36%) was sprayed to seedlings 24 hai (Table 2).

### Induction of ROS scavenging enzymes in *Pst* and FLBC treated leaves under glasshouse conditions

#### Effect of temporal changes on induction of defense enzymes

Greenhouse experiments were conducted to estimate the level of defense-related antioxidant enzymes in wheat seedlings inoculated with selected endophytic bacteria and CYR-31. The enzymatic activity was estimated at three different time points (24, 48, and 72 hai) in protective treatment.

#### SOD enzyme activity

Leaves of Inqilab-91, treated with FLBC formulation of 5 selected antagonist bacterial strains and CYR-31 race, resulted in a marked increase in SOD response (Fig. 5). In Inqilab-91, the maximum SOD activity was exhibited in *B. megaterium 6A* (6.97 ± 0.04) treated leaves, followed by *B. subtilus 11A* (6.09 ± 0.10) and *P. xylanexedens 7A* (5.02 ± 0.32) (Table 3).

**Table 3:** Relative induction of enzymes after bacterial and *Pst* inoculation in Inqilab-91 in protective treatment.

Bacterial treatment	SOD activity			POD activity			PPO activity			PAL activity			
	24 hai	48 hai	72 hai	24 hai	48 hai	72 hai	24 hai	48 hai	72 hai	24 hai		48 hai	72 hai
*Paenibacillus xylanexedens 7A*	1.05 ± 0.05a	1.03 ± 0.02a	5.02 ± 0.32a	9.47 ± 2.11a	22.13 ± 2.51a	33.63 ± 1.69a	2.40 ± 0.15a	2.05 ± 0.48a	1.63 ± 0.16a	96.10 ± 4.66a		98.93 ± 2.23a	75.88 ± 5.35a
*Serratia marcescens 3A*	1.00 ± 0.01a	1.03 ± 0.01ad	1.00 ± 0.01bf	7.93 ± 2.68ac	12.79 ± 1.95bc	29.51 ± 2.54a	0.90 ± 0.15ab	2.17 ± 0.62a	0.90 ± 0.14a	21.33 ± 3.81bf		45.84 ± 4.81b	49.18 ± 5.71bf
*Bacillus subtilus 11A*	0.82 ± 0.02b	0.93 ± 0.03be	6.09 ± 0.10cg	14.61 ± 1.62ad	17.17 ± 2.35acd	33.09 ± 3.84a	1.54 ± 0.48ab	4.32 ± 0.98bc	1.68 ± 1.13a	156.07 ± 9.78cg		153.33 ± 6.79c	105.12 ± 3.41cg
*Bacillus megaterium 6A*	0.91 ± 0.02c	0.98 ± 0.02ae	6.97 ± 0.04dg	18.94 ± 1.69bd	22.42 ± 2.35ade	44.14 ± 3.84b	4.18 ± 1.50ac	2.64 ± 0.33ac	1.58 ± 0.15a	140.24 ± 7.06dg		134.64 ± 5.66d	108.93 ± 2.06dg
*Staphylococcus agentis 15A*	1.03 ± 0.01a	0.95 ± 0.02ce	2.03 ± 0.09ef	7.93 ± 2.70ac	12.90 ± 1.71bdf	30.58 ± 3.54a	0.60 ± 0.15ab	3.21 ± 0.58ac	1.51 ± 0.38a	35.27 ± 8.02ef		69.23 ± 7.59e	55.71 ± 3.08ef

#### POD enzyme activity

The activity of POD was observed in co-inoculated leaves samples of wheat at different time intervals. It was observed that the Inqilab-91 leaves treated with *B. megaterium 6A* showed an increase in POD activity 44.14 ± 3.8 units change in absorbance min^−1^ g^−1^ of fresh tissue at 72 hai, as compared to other bacterial treatments. However, no significant difference in POD activity was observed in *P. xylanexedens 7A* and *B. subtilis 11A* (33.63 ± 1.69 and 33.09 ± 3.84) at 72 hai. The bacterial strain *S. agentis 15A* and *S. marcescens 3A* induced the 30.58 (± 3.54) and 29.51 (± 2.54) unit changes in absorbance min^−1^ g^−1^ of fresh tissue respectively at the 72 hai (Table 3).

#### PPO enzyme activity

The activity of PPO was different in all the bacterial treatments. In Inqilab-91, leaves treated with *Pst* and *B. subtilus 11A* showed maximum enzymatic activity (4.32 ± 0.98) at 48 hai, *B. megaterium 6A* (4.18 ± 1.50) at 24 hai followed by *S. agentis15A* (3.21 ± 0.58) at 48 hai. However, *S. marcescens 3A and S. agentis 15A* showed relatively low activity of PPO (2.17 ± 0.62) at 48 h which again declined at 72 hai (Table 3).

#### PAL enzyme activity

All bacterial treatments showed maximum induction of PAL activity at 24 and 48 h, after which it started decreasing at 72 h time point: the application of *B. subtilus 11A* (156.07 ± 9.78) and *B. megaterium 6A* (140.24 ± 7.06) at 24 hai. The enzyme activity was significantly high at 24 hai and then declined slowly in all treatments, except for *S. marcescens 3A* (49.18 ± 5.71), where maximum induction was observed at 72 hai (Table 3).

### Quantification of pathogenesis related proteins in the seedlings treated with FLBC and *Pst*

#### Expression pattern of antifungal gene (PR-1)

Expression analysis of the PR-1 gene in Inqilab-91, inoculated with CYR31 and selected bacterial strains, showed that the gene was induced in all five bacterial treatments. However, the induction was significantly higher in *P. xylenexedens 7A* (18.13 ± 1.55), *B. megaterium 6A* (16.07 ± 2.5), and* B. subtilus 11A* (14.88 ± 2.12) at all time points as compared to *S. marcescens 3A* (6.15 ± 1.17) and* S. agentis 15A* (2.80 ± 0.98). No significant difference was observed between *P. xylenexedens 7A, B. megaterium 6A, and B. subtilus 11A* at 48 and 72 hai, and between *B. megaterium 6A and B. subtilus 11A* at 24 hai (Table 4).

**Table 4:** Relative expression of Pathogenesis related protein genes in leaves of Inqilab-91 treated with selected bacteria and *Pst* in protective treatment.

Bacterial treatment	PR-1			PR-2			PR-4			PR-9			PR-10		
	24 hai	48 hai	72 hai	24 hai	48 hai	72 hai	24 hai	48 hai	72 hai	24 hai	48 hai	72 hai	24 hai	48 hai	72 hai
*Paenibacillus xylanexedens 7A*	3.62 ± 1.29 a	18.13 ± 1.55 a	16.34 ± 1.36 a	27.66 ± 1.61 a	35.38 ± 1.00 a	28.18 ± 0.67 a	9.49 ± 1.83 a	21.5 ± 2.67 a	21.71 ± 2.53 a	5.35 ± 1.47 a	13.16 ± 2.50 a	13.76 ± 2.68 a	0.64 ± 0.30 a	2.48 ± 1.13 a	6.69 ± 1.14 a
*Serratia marcescens 3A*	2.26 ± 0.80 ac	6.15 ± 1.17 b	3.19 ± 2.28 b	15.93 ± 1.47 a	17.64 ± 2.21 b	15.88 ± 3.56 b	1.46 ± 0.41 b	1.46 ± 0.12 b	10.1 ± 3.00 b	15.57 ± 4.34 a	22.01 ± 4.01 a	13.11 ± 0.31 a	1.82 ± 0.42 a	1.94 ± 0.63	0.96b ± 0.46 a
*Bacillus subtilus 11A*	3.71 ± 0.68 ab	14.89 ± 2.12 a	12.81 ± 2.46 a	59.39 ± 17.94 bc	76.56 ± 8.39 cd	72.5 ± 4.62 c	35.74 ± 4.67 c	23.29 ± 4.07 a	23.73 ± 1.55 a	72.81 ± 5.22 bc	80.23 ± 25.98 bc	25.55 ± 5.48 b	23.52 ± 2.11 b	13.00 ± 1.89 b	9.85 ± 2.00 c
*Bacillus megaterium 6A*	5.80 ± 0.34 b	13.77 ± 4.81 a	16.07 ± 0.61 a	55.46 ± 9.37 cb	74.08 ± 1.36 cd	81.92 ± 3.44 d	43.79 ± 2.63 d	46.05 ± 3.39 c	26.80 ± 5.64 a	68.33 ± 10.85 cb	67.57 ± 12.04 cb	44.96 ± 1.92 c	13.16 ± 2.76 c	5.86 ± 3.56 a	1.79 ± 0.52 db
*Staphylococcus agentis 15A*	1.03 ± 0.49 c	2.29 ± 0.27 c	2.80 ± 0.98 c	11.56 ± 0.78 a	7.44 ± 3.68 eb	8.85 ± 0.94 eb	3.44 ± 0.78 ab	1.27 ± 0.16 db	5.19 ± 0.41 cb	4.00 ± 1.47 a	16.37 ± 4.44 a	9.80 ± 2.74	0.82 ± 0.17 a	1.91 ± 0.52 a	1.24 ± 0.69 eb

Means were compared with Tukey HSD (p ≤ 0.05).

#### Expression pattern of *β*-1,3-glucanase activity (PR-2 gene)

Expression of β-1,3-glucanase PR-2 gene was induced in Inqilab-91, co-inoculated with CYR31 and 5 selected bacterial strains. With the bacterial priming of *B. subtilus 11A,* a relative expression of 76.56 ± 8.4 and with *P. xylanexedens 7A,* a relative expression of 35.37 ± 1.00 showed upregulation at 48 hai*. B. megaterium 6A* showed upregulation of the PR-2 gene with a relative expression of 81.9 ± 3.44 at 72 hai. Expression of PR-2 was significantly higher in *B. megaterium 6A* and *B. subtilus 11A* at all time points as compared to the other 3 bacterial treatments (Table 4).

#### Expression pattern of endochitinase activity (PR-4)

The PR-4 gene was induced in Inqilab-91, treated with CYR31, and bacterial strains. Expression of PR-4 was higher in *P. xylenexedens 7A, B. megaterium 6A,* and *B. subtilus 11A,* as compared to the other two bacterial treatments. However, the expression of PR-4 varied in bacterial treatments. Priming with *B. megaterium 6A* (43.79 ± 2.63) and (46.05 ± 3.392) exhibit maximum induction at 24 and 48 hai respectively, followed by *B. subtilus 11A* (35.74 ± 4.67) at 24 hai. Expression of PR-4 was significantly higher at 24 and 48 hai in the Inqilab-91 leaves treated with *B. megaterium 6A *(Table 4).

#### Expression pattern of peroxidase activity (PR-9)

In Inqilab-91, the expression of PR-9 was high in *B. subtilus 11A* treatment 72.81 ± 5.22 at 24 hai and 80.23 ± 25.98 at 48 hai. Similarly, in *B. megaterium 6A*, treatment of the expression of PR-9 was high i.e., 68.33 ± 10.85 at 24 hai and 67.57 ± 12.04 at 48 hai. The PR-9 gene was induced in all bacterial treatments, but the expression level was significantly low compared to *B. subtilus 11A* and *B. megaterium 6A* (Table 4).

#### Expression pattern of phenylalanine ammonia-lyase activity (PR-10)

The PR-10 gene was significantly induced at 24 hai in Inqilab-91 leaves, treated with *B. subtilus 11A* (23.52 ± 2.11) and *B. megaterium 6A* (13.16 ± 2.76). Although it remained induced at all time points, the expression decreased with an increase in time. Alternatively, PR-10 was induced in leaves of Inqilab-91 treated with other bacterial strains, but the level of expression was significantly low (Table 4).

## Discussion

The current study identified the bacterial endophytes that can be used as biocontrol agents (BCA) while using fungi-static and/or fungicide properties. The study also traced out the possible mechanisms that induce the defense system of wheat to control the stripe rust *Pst* with the help of endophytic bacteria*.*

Generally, PGPB reduces the disease by either killing the plant pathogen, by retarding its proliferation inside the host or indirectly strengthening the host cell immunity by minimizing the cell damage and pathogen progression through a mechanism of ISR (induced systemic resistance)^17^.

During the study, the 16 bacterial strains isolated from stripe rust-resistant cultivars were studied for their biocontrol potentials. Only 5 out of 16 bacteria showed promising results of controlling germination of stripe rust spores during in-vitro experiments. These 5 strains included *B. subtilis 11A, B. megaterium 6A, P. xylanexedens 7A, S. marcescens 3A*, and *S. agentis* 15A.

Two bacterial treatments (FLBC and FL) of these 5 selected strains were used to explore their biocontrol potentials. Out of these five bacteria, only three showed promising results in in-planta experiments. These treatments were applied to the leaves of a Pakistani wheat cultivar Inqilab-91 at 24 hbi and 24 hai. The results showed that the *Pst* disease incidence was significantly low in the cultivar treated with *B. subtilis 11A, B, megaterium 6A, P. xylanexedens 7A.*

Three strains (*Paneibacillus xylanexedens 7A, B. subtilis* 11A, and *B. megaterium* 6A) showing inhibitory effects belong to *Bacillus.* It is a well-established fact that the majority of bacterial antagonists belong to the *Bacillus*^18^*.* Bacilli not only replicate rapidly inside the host but also are resistant to adverse environmental conditions. They also have broad-spectrum biocontrol abilities. *B.* *subtilis* plays an important role in plant growth promotion and activation of a plant defense mechanism by triggering the induced systemic resistance (ISR) in plants^19^. Bacilli are also known to produce many active compounds with antimicrobials and antifungal properties, such as HCN, siderophores, and phytotoxins production, which can help the plant in its combat against the invading pathogen^20^. *B. subtilis* RC 218 and *B. amyloliquefaciens* FNL13 have been used for the biocontrol of *Fusarium* Head Blight^21^, while *B. subtilis* strain E1R-j and *B. subtilis* strain QST 713 have been used for the biocontrol of stripe rust and leaf rust of wheat^12,13^.

Among the two formulations, FLBC formulation of *B. subtilis* 11A, *B. megaterium* 6A, and* P. xylanexedens* 7A exhibited pronounced results. FLBC bacteria showed both curative effects and protective effects, while FL treatment of these bacteria showed only protective effects. In FL treatment, the reduction in disease severity confirmed the protective effect, which could be due to the release of metabolites in FL treatment. These metabolites included HCN, which is also known as a broad-spectrum antimicrobial compound, and has been reported for the suppression of disease in tobacco caused by *Thielaviopsis basicola*, cucumber caused by *Pythium ultimum*, and tomato caused by *Fusarium oxysporum* f. sp. *radicis-lycopersici*.^14,22^. Anand et al.^23^ reported reducing *P. infestance* mycelial growth by BCA producing HCN. The exact mechanism of how HCN reduces the growth is not yet confirmed. However, it is suggested that HCN might reduce the disease severity by inhibiting the growth of urediospores, which includes non-germinating spores or germinating spores with germ tubes less than spore radius and ruptured spores. In some cases, the germ tubes were also found damaged with the malformed morphology i.e., shrinkage or swelling. These findings were supported by SEM analysis of the treated leaf samples. Such abnormalities have also been observed and attributed to antifungal and chitin-lytic behavior of *S. marcescens* strain B2 when soil-borne pathogens *R. solani* and *F. oxysporum* were treated with it^24^*.* However, in FLBC treatment, reduced disease severity and change in reaction type were observed. The reduced disease severity was associated with competition between the bacterial cells and fungal spores for the site of entry and release of metabolites, which stopped the elongation of germ tubes. These bacteria also showed increased production of HCN and siderophore, which are well-known biocontrol agents.

The FLBC treatment of three bacteria (*Bacillus subtilis* 11A,* Bacillus megaterium* 6A*, and Paneibacillus xylanexedens* 7A) showed a change reaction type that was associated with inducing systematic resistance. No change in reaction type was observed in the plants treated with *S. marcescens* 3A and* S. agentis *15A*.* This fact can be linked with colonization efficiency. The bacteria that can colonize and reproduce inside the host can give out striking results. In our study, the colonization of inoculated bacteria in leaf tissues was also confirmed by RFLP analysis. However, keeping in view that FLBC treatment of these three bacterial strains resulted in a change in reaction type, and two bacteria strains result in reduce disease severity, two main characters of induced systematic resistance i.e., ROS production by measuring antioxidant enzyme activity and expression of pathogenicity related proteins were studied.

Production of reactive oxygen species (ROS) is typically a non-specific plant defense to induce hypersensitive response and program cell death against biotrophic pathogens, which need a living host for its survival^25^. To detoxify the initial burst of ROS produced by plants, the endophytic bacteria may produce ROS-scavenging enzymes. A high number and diversity of genes encoding ROS-scavenging enzymes are represented in the metagenome of the endophytic bacterial communities^26^.

In the present study, the wheat seedlings treated with endophytic bacteria also showed significantly reduced *Pst* development and moderately resistant phenotype. These seedlings also showed enhanced activity of antioxidant enzymes or ROS scavenging enzymes PAL, SOD, PO, and PPO associated with IR (induced resistance). Other studies have also shown oxidative burst in rice and Chinese medicine plant *Atractylodes lancea* after the colonization of endophytic bacteria^27–29^.

An increase in PAL activity was also observed when exposed to the bacterial strain in comparison with the untreated control^30^. The high phenolic activity was induced by the endophytic bacteria in wheat crop against the powdery mildew^31^. In plants, PAL plays an important role in the synthesis of phenolic compounds through the phenylpropanoid pathway^32^. PPO also activates plant defense against various plant pathogens. Genetically modified tomato plants, which were genetically modified for overproduction of PPO, were highly resistant against various pathogenic diseases^33^. PPO plays a crucial role in plant defense systems by catalyzing the oxidative reactions of phenolic compounds^34^, which are highly effective against fungal plant pathogens. Many members of *Bacillus* species were found to be highly active in these types of defense-eliciting activities.

The protein extracts collected from leaves of wheat cultivars, treated with both fungus and the bacterial strains, showed increased activity of POD and SOD.

*Bacillus cereus* AR156 on tomato (*L. esculentum*) under biotic stress showed upregulation of CAT, SOD, and POX in the host plant, probably assuming activation of the host defense system^35^. POD is the main precursor of lignin biosynthesis, so a rise in POD level may lead to higher lignin biosynthesis in rust-infected leaves. In cereal crops, induced cellular lignification proved to be an important mechanism in the establishment of disease resistance against many fungal pathogens, including stripe rust^36^. In the current study, the activity of the POD gene was high amongst the total protein extracted from leaves and plants treated with three bacterial cultures.

The other mechanism, by which endophytic bacteria can induce the resistance response in inoculated plants, is by activating the expression of pathogenesis-related protein genes. In the current study, increased production of PR protein genes in the leaves of cultivars inoculated with bacterial strains indicated that the change in the reaction type is also due to activation of PR protein genes. The expression of pathogenesis-related protein genes (PR-1, PR-2, PR-4, PR-9, and PR-10) was evaluated in the bacterial and fungal co-inoculated plants. PR-2 is a β-1,3-glucanase enzyme. High expression of this gene plays a major role in plant defense systems through callose deposition^37^. β-1,3-Glucanases hydrolyze β-1,3-glucans found in the fungal cell wall, which could lead to degradation of the fungal cell wall and interfere with its deposition. PR-4 is an endochitinase enzyme. Expression of this gene supports plant defenses by breaking bonds between the C1 and C4 of 2 consecutive N-acetylglucosamines of chitin, which is the main constituent of the fungal cell wall^38^. High expression of the PR-4 gene from wheat was shown to inhibit spore germination and hyphal growth in vitro assays^39^. PR-9 is a specific type of peroxidase that acts in cell wall ramification by catalyzing lignification, making it difficult for the pathogen to cross the barrier. PR-9 also enhances resistance against pathogens by producing reactive oxygen species (ROS)^40^. PR-10 encodes a ribonuclease (RNase)-like protein, but its exact functions are unclear^41^.

In cell signaling, salicylic acid and jasmonic acid/ethylene pathways are considered major signaling pathways. These two pathways are involved in the activation of the gene to produce PR protein. It is a well-documented fact that PR-1and PR-2 can be activated by SA application in wheat^42^, whereas jasmonate (JA) activates PR-3, PR-4, PR-9, and PR-10^43,]^^44^. Endophytic bacteria have the potential to produce salicylic acid and jasmonic acid hormones, thus the presence of the endophytes having the potential of producing these hormones might trigger both salicylic acid and jasmonic acid signaling pathways simultaneously in the plant^45–47^. The salicylic acid and jasmonic acid/ethylene pathways were primed simultaneously by *Bacillus cereus* AR156 in the *A. thaliana* plant. The activation of these pathways/defense genes gives rise to improved plant immunity and resistance to pathogenic microbial infection.

Other studies have also found that endophytic bacteria confer resistance to pathogens by various mechanisms. Endophytic actinobacteria isolated from wheat tissues up-regulate defense genes like PR-1 and PR-4 of systematic acquired resistance (SAR), as well as PR-1.2 and Hel genes of the jasmonic acid/ethylene (JA/ET) pathway in *A. thaliana*^48^.

Endophytic *Bacillus* spp. have also been reported to produce antifungal lipopeptides. These lipopeptides can induce host defense gene expressions like PR-1 (antifungal protein) and PR-4 (endochitinase) in maize^49^. These lipopeptides can trigger the expression of PR proteins in the host without interaction with pathogens. It is reported that plant growth-promoting *B. mycoides* strain Bac J and *B. pumilus* strains provide ISR in sugar beet by enhanced peroxidase (PR-9) activity, an increased production of one chitinase (PR-4), and two isozymes of β-1,3-glucanase^50^. Similarly, *Bacillus pumilus* INR7 is also reported to induce systemic resistance by triggering the expression of PR protein genes against *Xanthomonas axonopodis* in pepper^51^.

## Conclusions

The bacteria isolated from stripe rust-resistant cultivars showed the potential of inhibiting *Pst* urediospores germination, both in in-vitro and in planta experiments. The reduction in stripe rust disease severity in Pakistani wheat variety Inqilab-91 could be attributed to the increased activity of defense-related enzymes, antioxidant enzymes, and the expression of pathogenesis-related proteins. The three bacteria identified in this study showing the most promising results have great potential and can be used against other cultivars and stripe rust races for exploring their biocontrol potential. The whole-genome sequencing and metabolome analysis of these bacteria might help to identify the exact mechanism involved in the biocontrol of rust.

## Methods

The present study was undertaken in the Plant Biochemistry, Molecular Biology and Biotechnology Laboratory, COMSATS University, Islamabad, and Crop Disease Management Laboratory (College of Plant Protection, Northwest Agriculture and Forestry University Yangling, China. All the experiments were conducted in the accordance with the relevant institutional, national, and international guidelines/legislation.

### Wheat cultivars, stripe rust isolates, and bacterial strains

Inqilab-91 along with Morocco were used in the greenhouse experiments. Seeds of the cultivars were obtained from the National Agricultural Research Center Islamabad (Farrakh Mehboob Provided the seeds). Chinese Yellow Rust 31 (CYR31) race was used in the laboratory and in-planta experiments^52^. The bulk inoculum was used in semi-field conditions on Inqilab-91. Wheat plants were grown in a greenhouse at 25 °C with alternative light and dark periods^53^. A total of 16 bacterial strains previously isolated from stripe rust-resistant Pakistani cultivars^16^ were used to explore their biocontrol potentials.

### Multiplication of *Puccinia striiformis tritici* inoculum

A total of 15–30 seeds of susceptible wheat cultivar Morocco were sown in pots and then transferred to the seedling growth chamber, preset at a temperature of 18 °C. At the two leaves stage using the brushing technique, the leaves of susceptible cultivars were inoculated with the urediniospores of *Pst*. Inoculated plants were incubated in a dew chamber for about 1 day containing 100% humidity at 9 °C, and then transferred to a growth room already maintained at 17–18 °C (16/8 h)^54^. After 15 days of inoculation, infected leaves were gently brushed with a soft-bristled paintbrush, and urediniospores were collected in a glass tube, stored at 4 °C for three days, for drying before further examination. The excess urediniospores were stored in cryo-vial at − 80 °C.

### Preparation for bacterial formulations

The culture of bacterial strains was obtained by transferring a single colony into a 250 mL conical flask containing 100 mL lysogeny broth (LB) and constant shaking on an orbital shaker at 120 rpm for 48 h at 28 °C. Two types of formulations i.e., fermented liquid with bacterial cells (FLBC) and fermented liquid without bacterial cells (FL), were prepared. For the FLBC, the above-mentioned procedure of bacterial culturing was repeated. For the production of FL, the LB media with bacterial cells was centrifuged at 10,000×*g* at 4 °C for 20 min. The supernatant was collected as FL formulation^10^.

### Evaluation of inhibitory effects of bacterial isolates on germination of *Puccnia striiformis* urediniospores

The bacterial isolates were tested for their inhibitory effects on the germination of *Pst* urediniospores. Fresh urediniospores of stripe rust were evenly spread with camel hairbrush on water agar with bacterial culture (1 × 10^6^ CFU mL^−1^). A total of 150 spores were used in each treatment. Agar plates without bacterial culture were used as controls. Plates were kept at 9 °C in an incubator for 24 h. The germination and inhibition of urediniospores were checked using two bacterial formulations. Growth and germination of urediniospores were observed by an optical light microscope (LELICA DM LB2). The experiment was a completely randomized test design, with three technical replicates and two experimental replicates.

### Biocontrol potential of selected bacterial strains on urediniospores germination at the seedling stage under greenhouse conditions

Thirty (30) seeds of Inqilab-91 were sown in pots (10 cm wide at the top, 5 cm wide at the bottom, and 6 cm height) filled with a sterilized soil mixture (soil and nursery substrate, 3:1). Seven (7) days old seedlings were used for the assessment of curative and protective effects of selected bacterial strains. For the assessment of curative effects, seedlings were sprayed with FL and FLBC formulations 24 h after inoculation (hai). For the assessment of protective effects, 7 days old seedlings were sprayed 24 h before urediniospores inoculation (hbi). In both experiments, fresh urediniospores at the concentration of 3 × 10^4^ spores mL^−1^ along with FL and FLBC were used. The inoculated seedlings were kept in a specialized humidity chamber set at 10 °C for 24 h in the dark with relatively 100% humidity and then transferred to an already set greenhouse with a 16/8 h cycle (light/dark cycle) at 12–18 °C (dark period–light period). The control seedlings were sprayed with LB medium without any bacterial suspension. Disease assessments based on percent (%) of infected leaf surface (severity) and infection types (IT) were recorded 14 days post-inoculation until the control plants (Inqilab-91 inoculated with LB medium) reached maximum sporulation. IT data were recorded based on a 0–9 scale^55^. Infection types between 0 and 4 were considered as resistant (R), ITs between 4 and 6 as moderately resistant, and ITs between 7 and 9 were considered susceptible (S). Control efficacy was calculated using rust severity data following the procedure described by^12^. The experiment with the same treatments was conducted twice.

### Scanning electron microscope examination of *Pst* urediniospores after bacterial treatments

Wheat leaves of inoculated seedlings were sampled at 24 hai for scanning electron microscopic examination. The leaves were treated following the method described by^12^*.* Germination of urediniospores was observed using a JEOL-1230 SEM Scanning Electron Microscope.

### Bio-control effect of endophytic bacterial strains at adult plant under semi-field conditions

The antagonistic effect of bacterial strains on stripe rust urediniospores germination was tested in a pot experiment under semi-natural conditions during the 2017–2018 winter season. A pot trial was conducted on Inqilab-91 planted at the COMSATS University Islamabad, Pakistan. Fifteen seeds of Morocco and Inqilab-91 were planted in pots of 15-cm in diameter and 15-cm in height filled with potting soil mixture as described above. Sterilized soil was used in pots. Morocco (without the bacterial inoculum) was considered a positive control for the development of disease under natural conditions. Treatments were performed in a RCBD (randomized complete block design) of three replicates of each treatment.

Bulk inoculum of stripe rust pathogen was used. Inqilab-91 treated with *Pst* and bacterial formulations, without bacterial treatment, and with *Pst* inoculation were used to calculate disease control efficacy.

The two formulations (FL and FLBC) were sprayed onto the leaves at the flag leaf stage, 24 hai to determine the therapeutic effect and 24 hbi to determine the protective effect. The disease incidence was recorded when the positive control Morocco reached 100% susceptibility.

### Disease scoring data

Disease scoring data including rust severity (% of diseased leaf area) and infection type (IT) were recorded 14–15 days post *Pst* inoculation when the control was at maximum sporulation. The DS (Disease Severity score) on leaf surface was divided into six ratings: 0 = no infection/symptoms, 1 ≤ 5% infected, 3 = 6–25% infected, 5 = 26–50% infected, 7 = 51–75% infected, 9 = more than 75% infected^56^.

Control efficiency (CE) was calculated following the procedure of^12^. The experiment was performed in replicates and repeated twice.

### Temporal changes in induction of antioxidant enzymes

Antioxidant enzyme activity was estimated in leaves collected from the bacteria and *Pst* inoculated wheat seedlings. These enzymes included superoxide dismutase (SOD), phenylalanine de-aminase (PAL), polyphenol oxidase (PPO), and peroxidase (POD).

### Sample collection

The leaf samples of the above-treated seedlings were collected at different time points (24, 48, and 72 hai) from Inqilab-91. Randomly, 3–5 leaf samples were taken at each time point; leaf samples were mixed and divided into three replicates (groups). The samples were then wrapped in aluminum foil, quickly dipped into liquid nitrogen to freeze, and then shifted into the − 80 °C refrigerator for temporary preservation.

### Extraction of enzyme

Leaf samples (200 mg) of inoculated seedlings were grounded in liquid nitrogen, and the mixture was prepared by adding 2 mL of pre-cooled extraction buffer (0.1 M phosphoric acid buffer, pH 7.8 along with polyethylene pyrrolidone). The fully grounded mixture was centrifuged (13,000*g*) at 4 °C for 20 min. The supernatant was collected to determine the activity of superoxide dismutase (SOD), peroxidase (POD), phenylalanine de-aminase (PAL), and polyphenol oxidase (PPO). Total protein quantification was carried out using the Bradford^57^ method. The values regarding total protein contents were expressed as mg protein g^−1^ of fresh weight.

### Estimation of antioxidant enzymes activity

#### Superoxide dismutase (SOD) activity

For the determination of SOD activity, reaction mixture containing 50 mM potassium phosphate (pH 7.8), 14.5 M d-methionine, 2.5 mM NBT, 3 µM EDTA and 60 µM riboflavin was used. The tubes containing the reaction medium (1.0 mL) and protein extract (0.1 mL) were exposed to the fluorescent lamp for 15 min under 20 W. As a control, the reaction medium without protein sample was illuminated while the blank solution was kept in the dark. The optical density (OD) of each sample was taken at 560 nm in a spectrophotometer^58^. One unit of SOD enzyme was considered as “the amount of enzyme able to inhibit by 50% the photoreduction of NBT under the experimental conditions”. The SOD activity was expressed in U mg^−1^ of protein. The activity of the SOD enzyme was calculated by the following formula:

$${\text{SOD vigor }}\left( {{\text{U g}}^{{ - 1}} \;{\text{fw}}} \right) = \frac{{\left[ {\left( {{\text{control}} - {\text{sample}}} \right) \times {\text{1}}00\% \times {\text{ total volume of enzyme liquid }}\left( {{\text{mL}}} \right)} \right]}}{{\left[ {{\text{control}} \times {\text{5}}0\% \times 0.{\text{1 }}\left( {{\text{mL}}} \right) \times {\text{sample quality }}\left( {\text{g}} \right)} \right]}}$$

#### Peroxidase (POD) activity

POD activity was quantified in a spectrophotometer by oxidizing the 0.1 mL protein sample in 3 mL of 0.05 M phosphoric acid buffer, pH 5.5, 2 mL of 2% hydrogen peroxide (H_2_O_2_) as the oxidant, and 36 μL guaiacol as the hydrogen donor per 50 mL buffer. The oxidation of guaiacol was measured at 470 nm at 25 °C and was expressed in U g^−1^ FW min^−1^^59^.

Enzyme activity was defined as “an increase in absorbance of 1 unit enzyme at 470 nm min^−1^ at 25 °C”. The specific activity of POD was expressed in U mg protein^−1^.

The enzyme activity is calculated from the following formula:

$${\text{POD Vigor }}\left( {{\text{U g}}^{{ - 1}} \;{\text{fw}}\;{\text{min}}^{{ - {\text{1}}}} } \right) = \frac{{\left[ {\left( {{\text{a}}^{{{\text{initia}}}} - {\text{a}}^{{{\text{2min}}}} } \right) \times {\text{enzyme Liquid Total volume }}\left( {{\text{mL}}} \right)} \right]}}{{\left[ {{\text{2 }}\left( {{\text{min}}} \right) \times 0.{\text{1 }}\left( {{\text{mL}}} \right) \times {\text{sample quality }}\left( {\text{g}} \right)} \right]}}$$

#### Phenylalanine de-aminase (PAL) activity

Total 0.1 mL of protein extract was added in 0.1 M Phosphoric acid buffer (pH 8.8) and 1.0 mL of 20 mM l-phenylalanine. The mixture was incubated for 30 min at 37 °C. The reaction was stopped by adding 0.1 mL of HCl (0.6 M). PAL activity was determined based on the production of trans-cinnamate, by measuring the change in absorbance at 290 nm. The blank represented the crude protein mixed with l-phenylalanine with no incubation time^60^.

$${\text{Pal Vigor }}\left( {{\text{U g}}^{{ - 1}} {\text{ fw min}}^{{ - {\text{1}}}} } \right) = \frac{{\left[ {{\text{a}}^{{{\text{29}}0}} \times {\text{total volume of enzyme liquid }}\left( {{\text{mL}}} \right)} \right]}}{{\left[ {{\text{3}}0{\text{ }}\left( {{\text{min}}} \right) \times 0.0{\text{1}} \times 0.{\text{1 }}\left( {{\text{mL}}} \right) \times {\text{sample quality }}\left( {\text{g}} \right)} \right]}}$$

#### Polyphenol oxidase (PPO) activity

The PPO reaction mixture contained 0.1 mL of protein extract and a 3.0 mL substrate solution containing catechol (0.1 M) as the substrate and sodium phosphate (0.1 M) buffer (pH 7.4). The reference cuvette contained only the substrate. The rate of oxidation of catechol was monitored at 495 nm for 1 min at 25 °C. The enzyme activity was defined as an increase in absorbance of 0.001 min^−1^^60^.

### Quantification of pathogenesis related proteins (PR) genes

Quantification of pathogenesis-related proteins (PR) genes will be carried out from the sub-set of the same samples used for enzymatic assay collected at 24 hai, 48 hai, and 72 hai.

### RNA extraction and cDNA synthesis

RNA was extracted from treated leaves using the RNeasy Plant Mini Kit (Qiagen). RNA concentration was checked using a NanoDrop 2000 spectrophotometer (Thermo Scientific). To check the purity of RNA, 3 μL of RNA sample was run in a 2% agarose gel. cDNA was synthesized using 200 μg of RNA as a template. cDNA synthesis was carried out using the cDNA Synthesis Kit (Thermo Scientific). In PCR water, cDNA was diluted and 1:2 dilution of cDNA was used as the qRT-PCR template.

### Quantitative real-time PCR (qRT-PCR) for the expression of PR protein genes

The qRT-PCR reactions were executed for the target genes that encode the following PR protein genes, PR-1, PR-2, PR-4, PR-9, and PR-10. The primers were obtained from Shanghai Sangon Biological Engineering Technology & Services Company China (Table 5).

**Table 5:** Primer sequences of PR genes.

Target	Gene annotation	Forward primer	Reverse primer
PR-1	Antifungal	CAATAACCTCGGCGTCTTCATCAC	TTATTTACTCGCTCGGTCCCTCTG
PR-2	b-1,3-Glucanase	AAGCACTTTGGGCTGTTCAATCCG	CCAGGCAGCTTATTCGAACGCAAA
PR-4	Endochitinase	AAGTGCCTCCAGGTGACGAA	TGCACTGGTCGACGATCCT
PR-9	Peroxidase	CAAGGTGAACTCGTGATGGA	TTGAGGATTCAACCGTCGTT
PR-10	Ribonuclease	CAAGATGGTCGAGGCTTACC	CGAAGTCGATCATGAAGCAA
TaEF	Elongation factor	TTCGCCGTCCGTGATATGAGACAA	ATGCGTATATGGTGGTGGAGTGA

The final reaction volume was 12 μL having 1 μL of template cDNA, 5 μL of iTaq master mix (Biorad), 0.5 μM of primers, and PCR water. A negative control without a cDNA template was added to check the contamination as well as purity of the reagents. A Light Cycler 96 thermocycler (Roche) was used with the following thermal qPCR profile: 95 °C for 5 min; 95 °C 40 cycles of 10 s, 10 s at the appropriate annealing temperature (depending on primer used, 56–60 °C) and 72 °C for 10 s. The melting phase starts at 65 °C and is completed at 95 °C, with an increase of 1 °C in each step. The amplification of the product was visualized in 1% agarose gel. The specificity of the reaction was confirmed from the melt curve and the efficiency of each primer was checked using the standard curve. All reactions were carried out with three biological replicates. The relative gene expression levels were formulated by relative expression = 2^−ΔΔCq^^61^.

### Statistical analysis

Analysis of variance was performed on each data set, i.e., on germination and disease score. Mean urediniospores germination rate, the incidence of disease, disease severity, and control effect of different treatments were compared using the least significant difference (LSD) at p < 0.01, 0.05^62^.

Data were presented as means ± standard deviations (S.D.). Statistical differences between the different bacterial treatments and formulations were analyzed by analysis of variance (ANOVA). The mean differences were compared using a least significant difference (LSD) test. Differences were considered significant when *p* < 0.05. Each treatment was repeated twice with three replications. The experiment was conducted in triplicate. The statistical significance of up–down-regulation was further calculated with statistical software at *p* < 0.05. After normalization of the target with the reference gene, 0 value indicates that there was no difference in the gene expression level. For this study from the sequence of elongation factor (EF1) *Pst* specific primer (TaEF) was designed and used as a reference gene.
